# Overexpression of Pin1 and rho signaling partners correlates with metastatic behavior and poor recurrence-free survival of hepatocellular carcinoma patients

**DOI:** 10.1186/s12885-019-5919-3

**Published:** 2019-07-19

**Authors:** Lui Ng, Virginia Kwan, Ariel Chow, Thomas Chung-Cheung Yau, Ronnie Tung-Ping Poon, Roberta Pang, Wai-Lun Law

**Affiliations:** 1 Department of Surgery, Li Ka Shing Faculty of Medicine, The University of Hong Kong, Pok Fu Lam, Hong Kong; 2 Centre for Cancer Research, Li Ka Shing Faculty of Medicine, The University of Hong Kong, Pok Fu Lam, Hong Kong; 3 Department of Medicine, Li Ka Shing Faculty of Medicine, The University of Hong Kong, Pok Fu Lam, Hong Kong

**Keywords:** Pin1, RhoA, RhoC, Metastasis

## Abstract

**Background:**

Identification of molecular markers for early detection or prediction of metastasis is crucial for both management of HCC patient postoperative treatment and identify new therapeutic targets to inhibit HCC progression and metastasis. In the current study, we investigated the clinical correlation between Pin1, RhoA and RhoC and their association with HCC metastasis.

**Methods:**

Using a randomized study design of primary HCC samples from 139 patients, we determined messenger RNA expression of Pin1, RhoA and RhoC and their prognostic value.

**Results:**

Our findings demonstrated for the first time the clinical correlation of Pin1 in HCC metastasis. Pin1, RhoA and RhoC transcript levels were significantly higher in HCC specimens when compared with the paired adjacent non-tumorous liver. Pin1 overexpression was closely correlated with that of RhoA (R = 0.562, *p* < 0.001) and RhoC (R = 0.529, p < 0.001), and their co-overexpressions correlated with metastatic HCC (*p* = 0.000012) and poor recurrence-free survival of HCC patients (*p* < 0.00001), which showed better prognostic significance than either Pin1, RhoA or RhoC overexpression alone. Co-overexpressions of Pin1 + RhoA/RhoC were also an independent factor for predicting development of metastasis after curative resection in our multivariate regression model (*p* < 0.001).

**Conclusion:**

Pin1, RhoA and RhoC co-overexpressions are prognostic factor for metastatic HCC and predict poor recurrence-free survival.

**Electronic supplementary material:**

The online version of this article (10.1186/s12885-019-5919-3) contains supplementary material, which is available to authorized users.

## Background

Hepatocellular carcinoma (HCC) is a leading cause of cancer mortality worldwide, especially in Eastern Asian regions including Hong Kong and China where hepatitis B is more prevalent, while cases of HCC are also slightly increasing in low-incidence areas such as the United States and Canada [1]. Despite recent advances in therapeutic strategy and surgical techniques in treatment of primary cancer, the prognosis of HCC patients is still poor due to a high incidence of postoperative metastasis and recurrence [2]. Increasing researches have been focused on investigating the molecular pathways leading to cancer metastasis and the fundamental steps such as cell invasion and migration, in order to discover molecular markers for early detection or prediction of metastasis which is crucial for management of patient postoperative treatment, as well as to identify novel therapeutic targets to inhibit HCC progression and metastasis.

The identification and characterization of a peptidyl-prolyl cis/trans isomerase, Pin1, has led to the discovery of a new postphosphorylation regulatory mechanism in cell signaling pathways through promoting the *cis–trans* isomerization of specific proteins that are phosphorylated at Ser/Thr-Pro motifs [3–5]. Such conformational changes can have profound effects on the function of many Pin1 substrates. By this, Pin1 plays an important role in many cellular events, such as cell cycle progression, cell proliferation, and transcriptional regulation. In HCC, we have previously reported a high prevalence of Pin1 over-expression [6]. Our group and the others demonstrated that through interaction with different substrates, Pin1 induced cell proliferation, enhances cellular transformation and inhibited apoptosis in HCC [6–10].

The aim of this study was to investigate the clinical correlation between Pin1 and HCC metastasis. We also examined the potential interaction between Pin1 and two members of the Rho subfamily of the Ras superfamily of homologious genes, RhoA and RhoC, which has been implicated in tumorigenesis, tumor progression and metastasis of HCC [11–15] by determining the clinical correlation of their expressions individually and in combination with HCC prognosis. Our study will demonstrate the pivotal role of Pin1 in HCC metastasis and prognosis.

## Methods

### Patients and specimens

Fresh tumor specimens were randomly obtained from 139 patients (79 patients with paired tumor/non-tumor specimens and 60 patients with tumor specimens only) who underwent surgical resection of primary HCC at the Department of Surgery, Queen Mary Hospital, The University of Hong Kong, between 1998 and 2007. One hundred and four were men and thirty-five were women. Patients’ age ranged from 15 to 88 years, with a mean age of 58.6 years. The patient characteristics were summarized in Additional file 1: Table S1 and Additional file 2: Table S2. All samples were immediately frozen in liquid nitrogen and kept at -80°C until analysis. The study was approved by Institutional Review Board of the University of Hong Kong/Hospital Authority Hong Kong West Cluster (HKU/HA HKW IRB) and written informed consents were obtained from patients prior to their inclusion.

### RNA extraction and cDNA synthesis

Total RNA was extracted from tumors and their corresponding non-tumorous liver samples (if available) from HCC patients using Trizol reagent and RNA miniprep extraction kit according to the manufacture’s instruction (Life Technologies), and 500 ng each RNA sample was used to prepare cDNA using PrimeScript™ RT Master Mix (TaKaRa).

### Quantitative real-time polymerase chain reaction

Real-time PCR was performed in a final volume of 15 μl containing 1.5 μl RT transcript, 0.2 μM of each primer, 1X ROX reference dye and 7.5 μl of FastStart Universal SYBR Green Master (ROX) (Roche). The primers sequences were as follows: Pin1-FP: AAGATGGCGGACGAGGAG, Pin1-RP: CACTCAGTGCGGAGGATGAT; RhoA-FP: GTGCCCACAGTGTTTGAG, RhoA-RP: AGGGCTGTCGATGGAAAAA; RhoC-FP: GCAGGGCAGGAAGACTAT, RhoC-RP: GTTGGGGCAGAAGTGCTT; actin-FP: 5’- CGAGCATCCCCCAAAGTT-3’, actin-RP: 5’-GCACGAAGGCTCATCATT-3’. Real-time

PCR was carried out using the ABI 7900HT Fast Real-Time PCR System (Applied Biosystems) at 95°C for 10 min, followed by 40 cycles at 95 °C for 15 sec and at 56 °C for 1 min. The expression of actin was used to normalize that of the target genes. Each assay was done in triplicate, the average was calculated, and the expression level of targets mRNA was expressed as fold to actin..

### Statistical analysis

Data analysis was performed using SigmaStat 3.5 (Systat Software Inc., San Jose, CA, USA) or SPSS 16.0 (SPSS, Chicago, IL, USA). The Rank Sum test was used to analyze differences between experimental groups of clinical specimens. Spearman’s correlation test was applied to determine correlations. Chi-square test was used to compare categorized data. Disease-free survival (DFS) was calculated by the Kaplan-Meier method, and differences in survival rate were compared using the log-rank test. Univariate and multivariate analyses were performed using the Cox proportional hazards regression model. *P* < 0.05 was considered statistically significant.

## Results

### Overexpression of pin 1, rho a and rho C in HCC

**Fig. 1:**
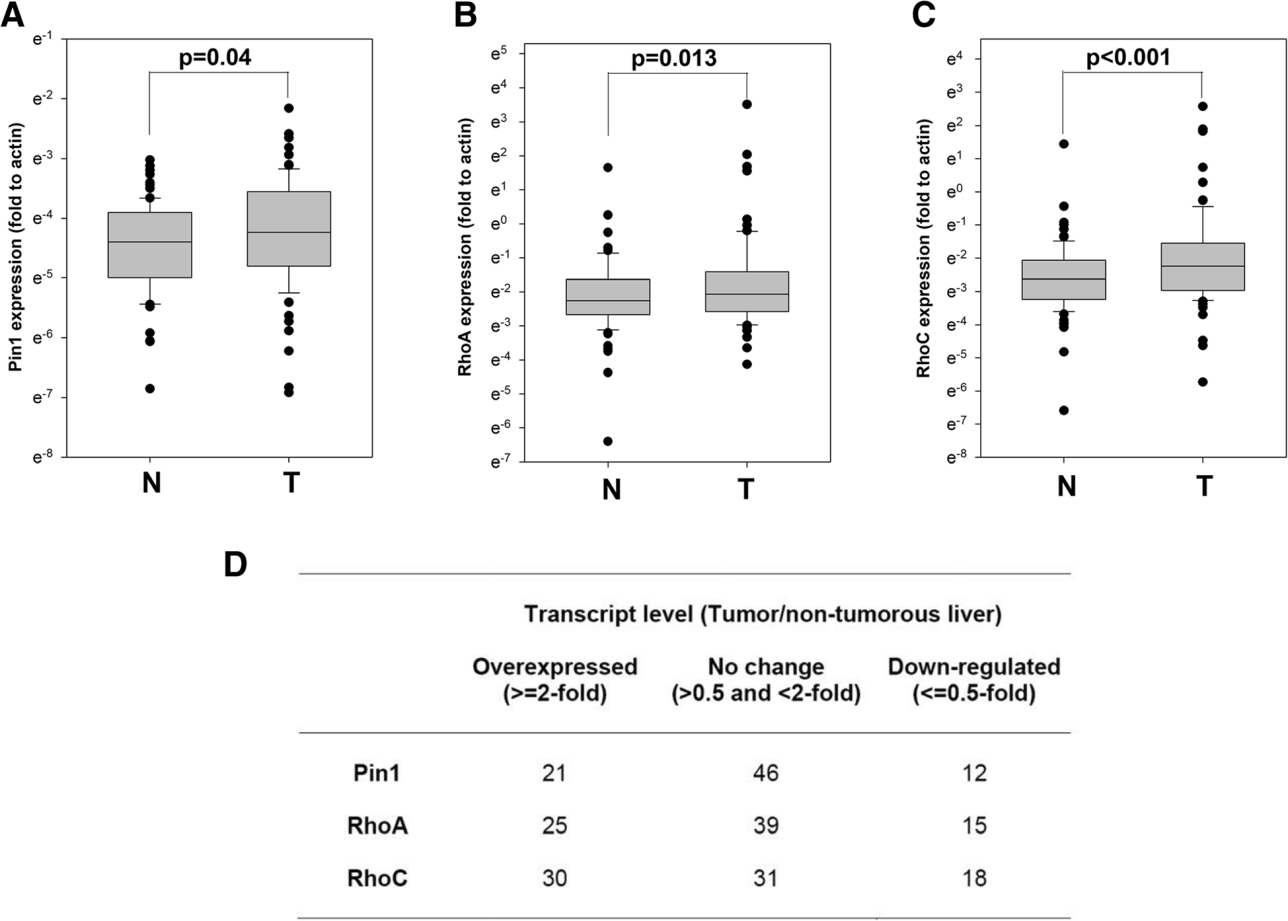
Expression of Pin1, RhoA and RhoC in HCC patients. The expression of Pin1 (**a**), RhoA (**b**) and RhoC (**c**) in *T* representing tumor tissue and *N* representing paired adjacent non-tumorous liver of HCC patients were calculated by the 2^-∆Ct (target gene-actin)^ method. Results are shown as box-plot representing the entire cohort. In the box-plot (first, third quartile, median), whiskers indicate maximum/minimum values and dots indicate outliers. (**d**), Statistics of Pin1, RhoA and RhoC expression in HCC patients, categorized into three groups (i.e. overexpressed, no change or downregulated) according to the expression in tumor tissue when compared with the paired adjacent non-tumorous liver

To study the role of Pin1, RhoA and RhoC in HCC carcinogenesis and progression, we first examined their transcript levels in 79 pairs of tumors and the adjacent non-tumorous liver of HCC patients by quantitative polymerase chain reaction, and expressed as the fold when compared with transcript level in the adjacent non-tumorous liver (Figure 1a-c). Pin1, RhoA and RhoC all showed significantly higher expression in HCC specimens (2.05, 3.58 and 5.39- fold, respectively), suggesting that they were in general overexpressed in HCC. We categorized the HCC patients into three groups according to the tumor to non-tumorous liver fold change of each of these transcripts (Figure 1d). Pin1, RhoA and RhoC were overexpressed in 21, 25 and 30 patients out of 79 patients, respectively, indicating these transcripts were frequently overexpressed in HCC.

We next examined the correlation of tumor level of Pin1, RhoA and RhoC transcripts with the clinicopathological parameters of the HCC patients. As shown in Table 1, their expressions were not correlated with gender, HBsAg status and cirrhosis. High expressions of Pin1, RhoA and RhoC were correlated with higher stage, presence of distant metastasis and recurrent disease. Interestingly, higher Pin1 and RhoC expression were significantly associated with smaller tumor, and high RhoA also demonstrated such trend, which might be due to dissemination of HCC cells from the primary tumor. Nevertheless, our clinical results demonstrated the close association of Pin1, RhoA and RhoC with metastatic HCC.

**Table 1:** Correlation between Pin1, RhoA and RhoC expression and clinicopathological parameters of the HCC patients in this study The median, lower quartile and upper quartile of the relative expression of each gene (fold to actin) of different experimental group were shown. Rank Sum test was performed to compare the difference

Gender	Male	Female	*p*-value
Pin1	0.039 (0.004–0.074)	0.023 (0.001–0.095)	0.317
RhoA	0.558 (0.101–1.264)	0.479 (0.121–1.675)	0.871
RhoC	0.182 (0.066–0.677)	0.314 (0.096–0.751)	0.364
Age	≤55	> 55	*p*-value
Pin1	0.004 (0.000–0.048)	0.058 (0.005–0.151)	<0.001
RhoA	0.189 (0.076–0.617)	0.941 (0.167–2.518)	< 0.001
RhoC	0.141 (0.056–0.355)	0.460 (0.129 0.897	<0.001
HBsAg	negative	positive	*p*-value
Pin1	0.058 (0.002–0.095)	0.020 (0.001–0.096)	0.284
RhoA	0.680 (0.121–1.936)	0.408 (0.117–1.567)	0.670
RhoC	0.356 (0.112–0.737)	0.255 (0.090–0.774)	0.966
Cirrhosis	negative	positive	*p*-value
Pin1	0.011 (0.001–0.075)	0.044 (0.003–0.096)	0.143
RhoA	0.346 (0.103–1.433)	0.790 (0.126–1.807)	0.308
RhoC	0.238 (0.095–0.760)	0.383 (0.086–0.654)	0.678
Tumor size	≤5	> 5	*p*-value
Pin1	0.045 (0.006–0.113)	0.008 (0.001–0.074)	0.021
RhoA	0.913 (0.189–1.654)	0.245 (0.099–1.354)	0.061
RhoC	0.433 (0.165–0.778)	0.187 (0.080–0.627)	0.048
Stage	low	high	*p*-value
Pin1	0.006 (0.001–0.061)	0.060 (0.018–0.152)	<0.001
RhoA	0.217 (0.101–0.944)	1.029 (0.306–2.750)	0.002
RhoC	0.187 (0.082–0.532)	0.470 (0.187–1.259)	0.003
Metastasis	absent	present	*p*-value
Pin1	0.006 (0.008–0.065)	0.065 (0.048–0.260)	<0.001
RhoA	0.210 (0.096–0.984)	1.746 (0.926–4.374)	<0.001
RhoC	0.188 (0.073–0.499)	0.763 (0.371–1.880)	<0.001
Recurrence	absent	present	*p*-value
Pin1	0.008 (0.001–0.051)	0.058 (0.003–0.150)	0.003
RhoA	0.189 (0.094–0.758)	1.137 (0.306–3.301)	<0.001
RhoC	0.132 (0.064–0.436)	0.501 (0.207–1.377)	<0.001

### Correlation between Pin1, RhoA and RhoC expressions and clinicopathologic features

**Fig. 2:**
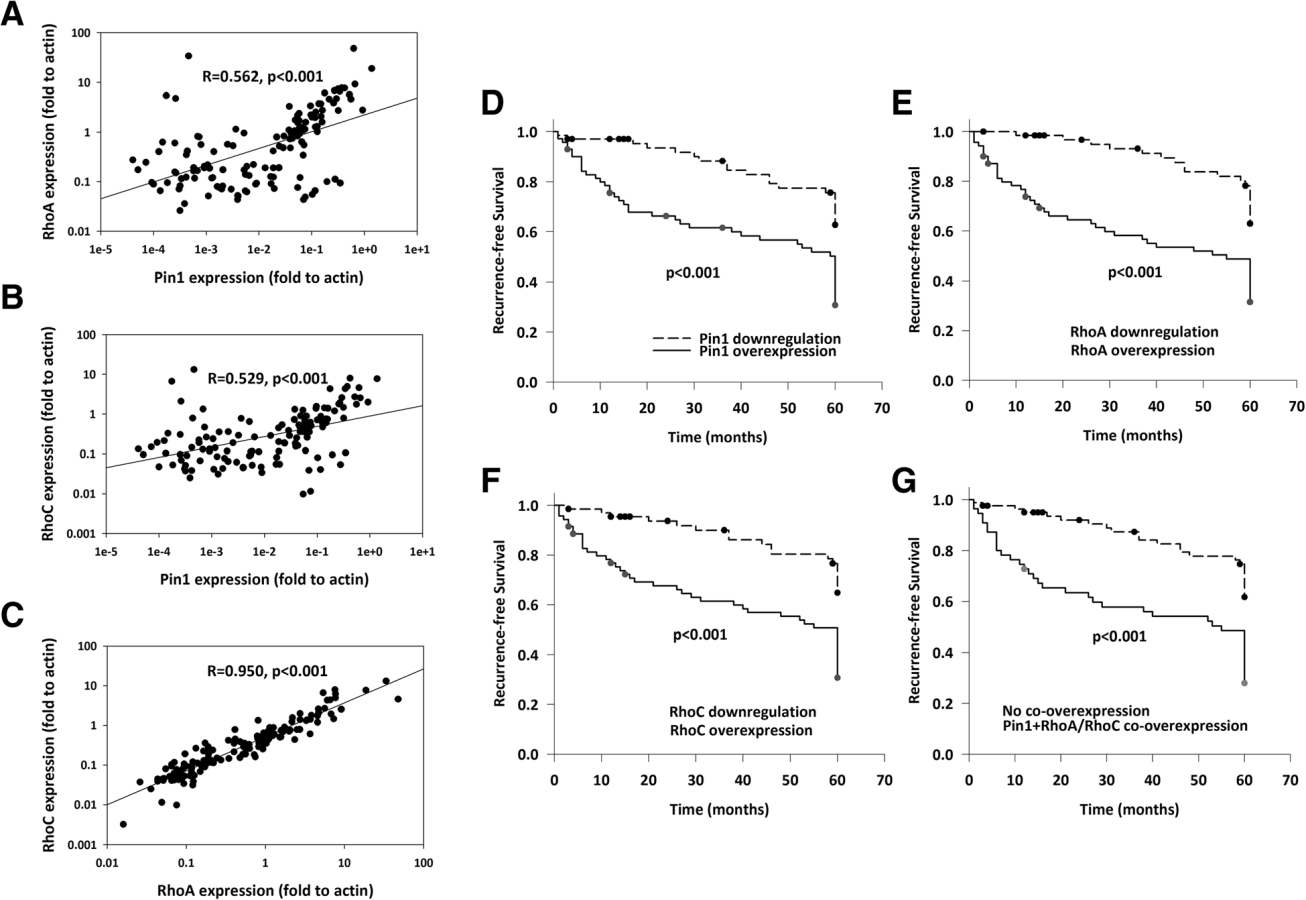
Correlative analysis of Pin1, RhoA and RhoC expression in HCC patients. Correlations of Pin1 with RhoA (**a**), Pin1 with RhoC (**b**), and RhoA with RhoC (**c**) as scatter-plot representations. Correlations of overexpressions of Pin1 (**d**), RhoA (**e**), RhoC (**f**), and Pin1/RhoA/RhoC co-overexpressions (**g**) with recurrence-free survival in HCC patients. Kaplan-Meier plots showed that downregulaiton of either one of them display a trend of longer disease-free survival, whereas their co-overexpressions was significantly associated with poor recurrence-free survival. The *p*-value was generated using log-rank test

Pin1, RhoA and RhoC showed apparently higher expression in HCC with higher stage and recurrence (Table 1), we hypothesized that their co-expressions were associated with these metastatic behaviors. To test our hypothesis, we first correlated the expressions of Pin1, RhoA and RhoC in HCC patients (Figures 2a-c). The results showed that expression of Pin1 was significantly correlated with RhoA (R=0.562, p<0.001) and RhoC (R=0.529, p<0.001), whereas RhoA and RhoC showed an even stronger correlation (R=0.950, p<0.001). Their close correlations supported our proposition that Pin1 associated with HCC metastasis through the action of RhoA and RhoC.

We next examined the association of Pin1, RhoA and RhoC co-expressions with stage. Overexpression of at least one of them were significantly correlated with higher stage (Table 2), and the association was stronger than that observed with Pin1, RhoA or RhoC alone as indicated by the lower p-value. In addition, Pin 1 and RhoA/RhoC co-overexpressions were significantly associated with metastasis (p=0.000012) and the association was again stronger than individual overexpression of Pin1, RhoA or RhoC (Table 3). Furthermore, their co-overexpression revealed significant correlation with recurrence (p<0.00001) when compared with overexpression of Pin1 (p=0.000135), RhoA (p=0.000044) or RhoC (p=0.00002) alone (Table 4). These results demonstrated that co-overexpressions of Pin1, RhoA/RhoC were crucial factors for HCC metastasis and recurrence.

**Table 2:** Clinical correlation of Pin1, RhoA and RhoC expressions with stage of HCC patients using Fisher exact test

	High (III-IV)	Low (I-II)	*p*-value
Pin1 expression			
Overexpression	36	36	
Downregulation	14	14	0.000094
RhoA expression			
Overexpression	33	32	
Downregulation	17	47	0.00478
RhoC expression			
Overexpression	34	31	
Downregulation	16	48	0.001458
Pin1/RhoA/RhoC expression			
At least 1 overexpression	43	39	
All downregulation	7	40	0.000025

**Table 3:** Clinical correlation of Pin1, RhoA and RhoC expressions with HCC metastasis using Fisher exact test

	High (III-IV)	Low (I-II)	*p*-value
Pin1 expression			
Overexpression	28	42	
Downregulation	6	62	0.000021
RhoA expression			
Overexpression	27	41	
Downregulation	7	63	0.000051
RhoC expression			
Overexpression	27	42	
Downregulation	7	61	0.000094
Pin1 + RhoA/RhoC co-expressions			
Co-overexpression of Pin1 + RhoA/RhoC	26	31	
No co-overexpression	8	73	0.000012

**Table 4:** Clinical correlation of Pin1, RhoA and RhoC expressions with recurrence of HCC patients using Fisher exact test

	Presence	Absence	*p*-value
Pin1 expression			
Overexpression	44	22	
Downregulation	25	48	0.000135
RhoA expression			
Overexpression	44	21	
Downregulation	24	49	0.000044
RhoC expression			
Overexpression	45	20	
Downregulation	24	49	0.00002
Pin1 + RhoA/RhoC co-expressions			
Co-overexpression of Pin1 + RhoA/RhoC	43	21	
No co-overexpression	18	55	<0.00001

### Effect of Pin1/RhoA/RhoC expression on recurrence-free survival

In order to identify patients with higher potential to develop future distant metastasis, we compared the prognosis of HCC patients with and without overexpression of Pin1, RhoA or RhoC, as well as those with and without co-overexpression of these three genes. As shown in Figures 2d-g, downregulation of Pin1, RhoA or RhoC displayed signficantly better recurrence-free survival. Moreover, patients with co-overexpressions of Pin1, RhoA and RhoC showed a significantly poor recurrence-free survival (p<0.001)..

A univariate Cox regression analysis was applied to identify important prognostic factors of recurrence-free survival, which tested parameters including age, gender, HBsAg status, cirrhosis, tumor size, TNM stage and Pin1/RhoA/RhoC co-expressions (Table 5). High stage and co-overexpressions of Pin1+RhoA/RhoC were identified as important risk factors for recurrence-free survival (p<0.001). Next, we generated a multivariate analysis using Cox regression model, which againshowed that higher stageand no co-overexpressions of Pin1+RhoA/RhoC were independent factors for the recurrence-free survival of HCC patients (p<0.001, Table 5). These results demonstrated that co-overexpressions of Pin1+RhoA/RhoC was an unfavorable prognostic factor for recurrence-free survival of HCC patients, and potentiated these genes as molecular therapeutic targets to combat HCC metastasis.

**Table 5:** Univariate and multivariate analyses showing that Pin1/RhoA/RhoC co- overexpression could serve as an independent prognostic factor for recurrence of HCC patients

	Relative risk (95% confidence interval	*P*-value
Univariate analysis		
Age, year (> 55 versus ≤55)	1.350 (0.802–2.270)	0.258
Gender (male versus female)	1.041 (0.576–1.881)	0.894
HBsAg (positive versus negative)	0.820 (0.451–1.489)	0.513
Cirrhosis (yes versus no)	0.921 (0.565–1.503)	0.743
Tumor Size (> 5 versus ≤5)	1.576 (0.950–2.614)	0.078
TNM stage (III to IV versus I to II)	4.857 (2.841–8.303)	<0.001
Pin1 + RhoA/RhoC co-overexpression (yes versus no)	2.804 (1.686–4.663)	<0.001
Multivariate analysis		
Age, year (> 55 versus ≤55)	0.749 (0.3359–1.564)	0.442
Gender (male versus female)	0.838 (0.433–1.622)	0.600
HBsAg (positive versus negative)	0.958 (0.495–1.854)	0.899
Cirrhosis (yes versus no)	0.890 (0.498–1.590)	0.694
Tumor Size (> 5 versus ≤5)	1.268 (0.692–2.326)	0.442
TNM stage (III to IV versus I to II)	3.453 (1.814–6.573)	<0.001
Pin1 + RhoA/RhoC co-overexpression (yes versus no)	2.159 (1.078–4.325)	<0.001

## Discussion

Development of metastasis accounts for the extremely poor prognosis of HCC patients. Therefore, an important part of HCC researches is the identification of crucial factors for HCC metastasis, which are potential biomarkers to predict the prognosis of patients, or even novel therapeutic target to combat HCC metastasis. Our team and other groups have previously reported the roles of Pin1 in HCC carcinogenesis and its clinical correlation with HCC oncogenesis (6-10), yet its association with metastasis has not been demonstrated in HCC thus far. On the other hand, RhoA and RhoC overexpressions have been correlated with HCC progression and metastasis in many clinical studies [11–16]. In this study, we aim to examine the correlation between HCC metastasis and expressions of Pin1, RhoA and RhoC, and further determine the clinical significance of their co-expressions.

This study demonstrates for the first time the correlation of Pin1, RhoA and RhoC with the development of metastasis in HCC patients. We found that HCC patients with overexpression of Pin1, RhoA or RhoC showed increased rate of metastasis, when compared with those with down-regulation counterparts. For example, around 40% of HCC patients with overexpression of Pin1, RhoA or RhoC developed distant metastasis, whereas the percentage decreased to around 10% in patients with down-regulation of Pin1, RhoA or RhoC, suggesting that their down-regulations impaired the metastatic processes. In addition, we further demonstrated that 46% of the patients with co-overexpressions of Pin1+RhoA/RhoC developed distant metastasis, while less than 10% patients with no such co-overexpressions developed metastasis. Similar trends were also observed in the recurrence-free survival analyses of HCC patients, in which patients with downregulations of Pin1, RhoA or RhoC showed a trend of better prognosis for recurrence-free survival when compared with those with overexpressions, and those with co-overexpressions of Pin1+RhoA/RhoC demonstrated a significantly poor recurrence-free survival than those with no co-overexpressions. Hence, our results clearly demonstrated the correlations of Pin1 with RhoA and RhoC as well as their involvement in the metastatic process, and their co-overexpressions associated with metastatic HCC, which further potentiates the development of combined inhibitors against Pin1/RhoA/RhoC in order to combat metastasis in HCC patients.

There are two possible linkages between Pin1 and RhoA/RhoC. Our previous study on Pin1 demonstrated a preferential Pin1 overexpression in HBV-related tumors [9], whereas other RhoC studies showed that HBV proteins including HBx and HBs enhanced the promoter activity of RhoC *in vitro* through upregulating the transcription factor Ets-1, resulting in overexpression of RhoC mRNA and proteins [17, 18]. In accordance, our results in this report revealed that HCC patients with HBsAg showed a significantly stronger overexpression of Pin1 and RhoC, and similar trend for RhoA, in HCC when compared to adjacent normal liver (Additional file 3: Figure S1), though the effect of HBV proteins on their transcription has not been demonstrated yet. Moreover, we previously demonstrated that interaction and binding of Pin1 to phosphorylated Ser41-Pro motif of HBx, followed by cis–trans isomerization and stabilization, significantly augmented the expression of HBx downstream target genes [9]. Hence we believed that Pin1 could regulate the expression of RhoA/RhoC through this pathway, as we observed a strong correlation between Pin1 and RhoA/RhoC expressions. In addition to upregulation of the RhoA/RhoC transcription level, Pin1 is also likely to affect the activity of RhoA/RhoC indirectly through its effect on HBx protein which activates RhoA *in vitro* [19], as well as directly or indirectly interacts with RhoA and RhoC and regulates activation, as we had confirmed the presence of interaction between Pin1 and RhoA/RhoC in our co-immunoprecipitation experiment (our unpublished results). Further experiments are warranted and still in progress to investigate the effect of Pin1 on the functional roles and molecular mechanism of RhoA and RhoC in HCC metastasis *in vitro* and *in vivo*.

In this study, we showed that the combination of biomarkers demonstrated a better prognostic value than either one of Pin1, RhoA or RhoC, suggesting that these factors act together in the cancer metastatic process and thus effect of downregulation of one factor could be compensated by the others. Therefore, it will be more comprehensive to monitor a combination of factors in this instance. Indeed, there are increasing evidences demonstrating the efficacy of combination of molecularly-associated biomarkers. For example, combination of osteopontin and its receptor CD44v6 improves the sensitivity and predictive range for predicting tumor recurrence and survival in non-small cell lung cancer patients [20], combination of hypoxia-induced autophagy- related gene Beclin1 and HIF-1α refine distant metastasis risk and predict poor prognosis of ER- positive, HER2-negative breast cancer [21],and miR-34a silencing in combination with its target genes c-Met and β-catenin predicts distant metastasis of colon cancer [22]. The results obtained in this and the above studies, demonstrated that a set of markers provided a better monitoring of the tumor cellular process which was associated with a more precise prognosis of the tumor condition, as well as identified potential targets to inhibit the progression of tumor or development of metastasis.

## Conclusion

To summarize, this study demonstrated for the first time the clinical correlation of Pin1 in HCC metastasis. Pin1 was significantly correlated with the transcript levels of RhoA and RhoC, and their co-overexpressions correlated with metastatic HCC and recurrence of HCC patients, suggesting that Pin1, RhoA and RhoC not only could be used as potential biomarker for predicting development of metastasis after curative resection, but also as potential therapeutic targets to inhibit HCC metastasis.

## Additional files

Additional file 1: **Table S1.** Patient characteristics in this study. (PDF 30 kb)

Additional file 2: **Table S2.** Pin1, RhoA and RhoC expressions (fold to actin) in all HCC samples and the patient’s clinicopathological parameters. (XLSX 25 kb)

Additional file 3: **Figure S1.** Comparison of Pin1, RhoA and RhoC overexpressions (Tumor/non-tumor) in HCC patients with or without HBsAg. (TIF 2557 kb)

## Data Availability

All data generated or analyzed during this study are included in this published article [and its supplementary information files].

## Abbreviations

HCC: Hepatocellular carcinoma
N: Adjacent non-tumor liver
Pin1: Peptidyl-prolyl cis-trans isomerase NIMA-interacting 1
RhoA: Ras homolog gene family, member A
RhoC: Ras homolog gene family, member C
T: Tumor
