# Supplementary material for: Overexpression of Pin1 and rho signaling partners correlates with metastatic behavior and poor recurrence-free survival of hepatocellular carcinoma patients
Source: BMC Cancer. 2019 Jul 19;19:713. doi: 10.1186/s12885-019-5919-3 (PMC6642482; doi:10.1186/s12885-019-5919-3)
Supplement: Supplementary file 1 — Table S1. Patient characteristics in this study. (PDF 30 kb) [file 12885_2019_5919_MOESM1_ESM.pdf]

Supplementary table 1. Patient characteristics in this study

Gender	Male	Female
	104	35
**Age**	**≤55**	**>55**
	55	83
**HBsAg**	**negative**	**positive**
	27	106
**Cirrhosis**	**negative**	**positive**
	76	61
**Tumor size**	**≤5**	**>5**
	55	82
**Stage**	**low**	**high**
	49	79
**Metastasis**	**absent**	**present**
	104	35
**Recurrence**	**absent**	**present**
	73	66

Note: The total number is less than 139 in some categories due to incomplete patient's information
