# Supplementary figures and images for: Overexpression of Pin1 and rho signaling partners correlates with metastatic behavior and poor recurrence-free survival of hepatocellular carcinoma patients
Source: BMC Cancer. 2019 Jul 19;19:713. doi: 10.1186/s12885-019-5919-3 (PMC6642482; doi:10.1186/s12885-019-5919-3)

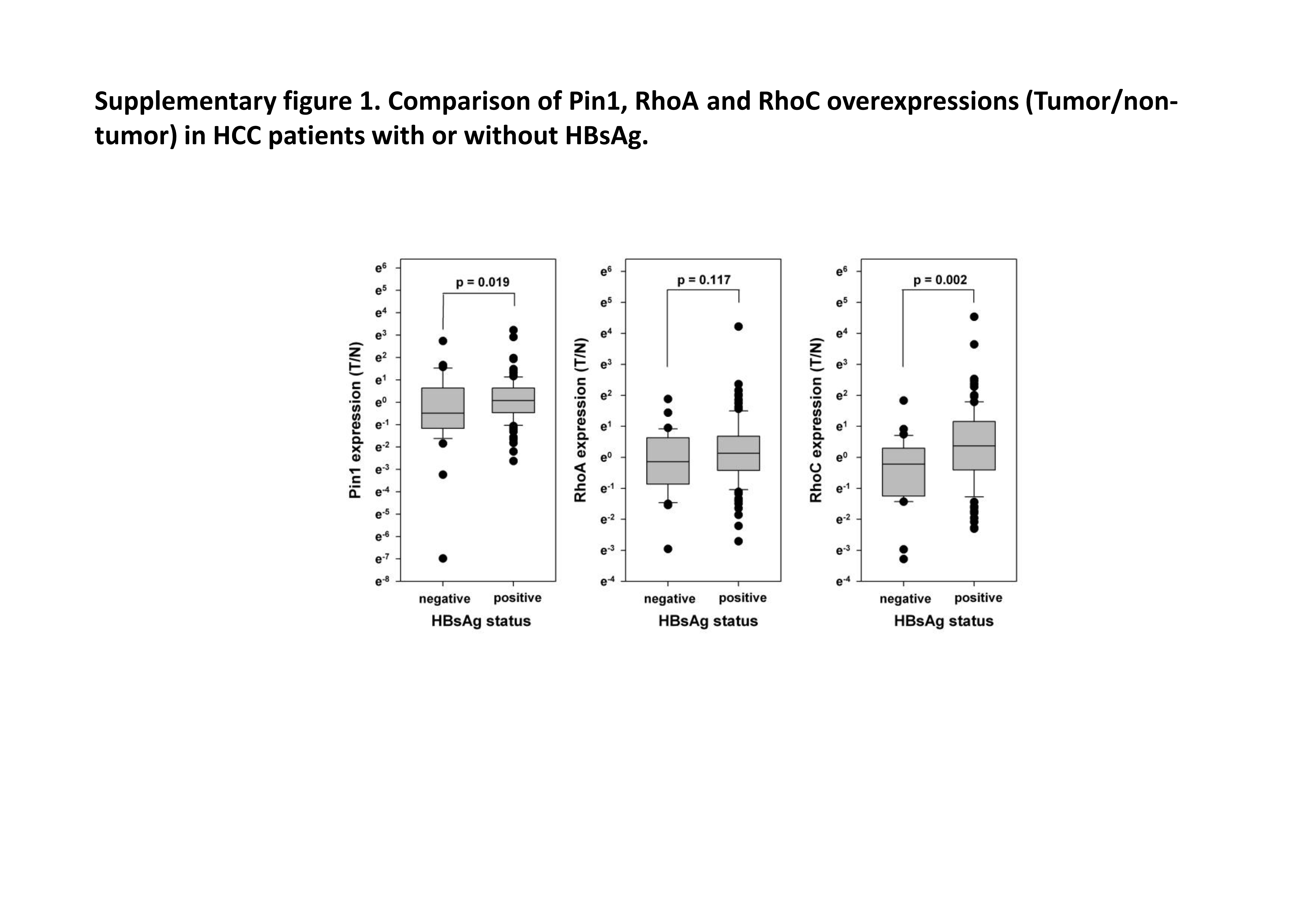

Supplement: Supplementary file 3 — Figure S1. Comparison of Pin1, RhoA and RhoC overexpressions (Tumor/non-tumor) in HCC patients with or without HBsAg. (TIF 2557 kb) [file 12885_2019_5919_MOESM3_ESM.tif]
